# Cutaneous Mechanoreceptor Feedback from the Hand and Foot Can Modulate Muscle Sympathetic Nerve Activity

**DOI:** 10.3389/fnins.2016.00568

**Published:** 2016-12-08

**Authors:** Nicholas D. J. Strzalkowski, Anthony V. Incognito, Leah R. Bent, Philip J. Millar

**Affiliations:** ^1^Department of Human Health and Nutritional Science, University of GuelphGuelph, ON, Canada; ^2^Toronto General Research Institute, Toronto General HospitalToronto, ON, Canada

**Keywords:** microneurography, muscle sympathetic neural activity, cutaneous, afferent feedback, autonomic nervous system, vibration

## Abstract

Stimulation of high threshold mechanical nociceptors on the skin can modulate efferent sympathetic outflow. Whether low threshold mechanoreceptors from glabrous skin are similarly capable of modulating autonomic outflow is unclear. Therefore, the purpose of this study was to examine the effects of cutaneous afferent feedback from the hand palm and foot sole on efferent muscle sympathetic nerve activity (MSNA). Fifteen healthy young participants (9 male; 25 ± 3 years [range: 22–29]) underwent microneurographic recording of multi-unit MSNA from the right fibular nerve during 2 min of baseline and 2 min of mechanical vibration (150 Hz, 220 μm peak-to-peak) applied to the left hand or foot. Each participant completed three trials of both hand and foot stimulation, each separated by 5 min. MSNA burst frequency decreased similarly during the 2 min of both hand (20.8 ± 8.9 vs. 19.3 ± 8.6 bursts/minute [Δ −8%], *p* = 0.035) and foot (21.0 ± 8.3 vs. 19.5 ± 8.3 bursts/minute [Δ −8%], *p* = 0.048) vibration but did not alter normalized mean burst amplitude or area (All *p* > 0.05). Larger reductions in burst frequency were observed during the first 10 s (onset) of both hand (20.8 ± 8.9 vs. 17.0 ± 10.4 [Δ −25%], *p* < 0.001) and foot (21.0 ± 8.3 vs. 18.3 ± 9.4 [Δ −16%], *p* = 0.035) vibration, in parallel with decreases in normalized mean burst amplitude (hand: 0.45 ± 0.06 vs. 0.36 ± 0.14% [Δ −19%], *p* = 0.03; foot: 0.47 ± 0.07 vs. 0.34 ± 0.19% [Δ −27%], *p* = 0.02) and normalized mean burst area (hand: 0.42 ± 0.05 vs. 0.32 ± 0.12% [Δ −25%], *p* = 0.003; foot: 0.47 ± 0.05 vs. 0.34 ± 0.16% [Δ −28%], *p* = 0.01). These results demonstrate that tactile feedback from the hands and feet can influence efferent sympathetic outflow to skeletal muscle.

## Introduction

The skin represents a complex organ innervated by a variety of sensory neurons (Zimmerman et al., 2014). Glabrous skin on the palms of the hand and soles of the feet are innervated by four classes of low threshold mechanoreceptors that convey tactile feedback to the central nervous system via large diameter Aβ myelinated afferents (Abraira and Ginty, 2013). Each class of cutaneous afferent is tuned uniquely to encode different features of non-noxious mechanical stimuli that together mediate the sense of touch (Strzalkowski et al., 2015a; Yau et al., 2016). Tactile feedback from the hands and feet are important for sensorimotor control (Kavounoudias et al., 1998; Zimmerman et al., 2014) and can be coupled to motoneuron activation (Fallon et al., 2005; Bent and Lowrey, 2013). Whether such afferent feedback from the skin is also capable of modulating efferent autonomic outflow is unclear. Low threshold mechanoreceptor afferent feedback from glabrous skin on the feet is critical for maintaining posture and balance (Kavounoudias et al., 1998) but could also serve as an afferent mechanism to trigger, for example, efferent sympathetic activation to help defend against the cardiovascular effects of orthostasis.

Clear evidence exists that stimulation of high threshold skin mechanoreceptors sensitive to noxious stimuli can influence heart rate, blood pressure, and efferent sympathetic outflow to skeletal muscle (Burton et al., 2016). Although pain and touch are known to be intricately related (Abraira and Ginty, 2013), the relationship between low threshold cutaneous mechanoreceptor afferent feedback and reflex efferent sympathetic (or parasympathetic) activity is not well-studied. Prior investigations have relied primarily on non-noxious electrical cutaneous stimulation to test the influence of cutaneous mechanoreceptor afferent feedback on blood pressure and muscle sympathetic nerve activity (MSNA). These studies demonstrate the capacity of electrical stimuli to modulate mean arterial pressure and/or MSNA (Hollman and Morgan, 1997; Donadio et al., 2002a,b; Gray et al., 2009; Labrunée et al., 2013), as well as highlight the central integration of somatosensory and baroreceptor inputs (Gray et al., 2009). However, the interpretation of results is limited by the observation that electrical stimulation can evoke arousal responses that also modulate efferent peripheral sympathetic activity in a similar manner as flashing light (i.e., not dependent on cutaneous mechanoreceptor afferent activity; Donadio et al., 2002a). Cutaneous electrical stimulation can also activate muscle (Goswami et al., 2012) and pain (Nordin and Fagius, 1995) afferents, limiting its use as a tool to understand the selective influence of low threshold mechanoreceptor feedback on cardiovascular control.

Applications of mechanical vibration are a commonly used technique to activate low threshold cutaneous afferents (Johansson et al., 1982; Ribot-Ciscar et al., 1989; Gandhi et al., 2011; Mildren et al., 2016). Studies employing microneurographic recordings of single cutaneous afferents demonstrate robust discharge responsiveness to vibration when applied over their receptive fields (Johansson et al., 1982; Trulsson, 2001; Lowrey et al., 2013). Cutaneous afferent classes are each tuned to narrow ranges of vibration stimuli, however, at large amplitudes all classes respond with different capacities (Johansson et al., 1982). Mechanical vibrations are inherent in natural stimuli (Bensmaia and Hollins, 2003; James et al., 2014), and experimentally applied vibration provides a controllable stimulus to selectively enhance low threshold mechanoreceptor afferent feedback. The efferent sympathetic response to the vibration of glabrous skin has not been studied.

Therefore, the purpose of the present study was to investigate whether low threshold cutaneous mechanoreceptor feedback from glabrous skin on the hand or foot is capable of modulating peripheral sympathetic outflow to skeletal muscle (MSNA). Given prior evidence for central integration of somatosensory and baroreceptive afferents (Gray et al., 2009; Goswami et al., 2011), our primary hypothesis is that the application of a cutaneous vibration stimulus above perceptual threshold would alter direct measurements of MSNA burst frequency. Further, as stimulation of group III/IV skeletal muscle afferents has been shown to elicit significant but brief alterations in MSNA burst frequency (Donadio et al., 2002b; Cui et al., 2006), our secondary hypothesis is that the largest sympathetic responses would occur during the onset of vibration.

## Materials and methods

### Ethical approval and participants

Fifteen healthy young normotensive participants (9 male; 25 ± 3 years [range 22–29 years]; 100 ± 8/63 ± 8 mmHg) were recruited from the University of Guelph student population. None of the participants had any known neurological, musculoskeletal, or cardiovascular disorders, and all possessed a body mass index <30 kg/m^2^. This study was approved by the University of Guelph Research Ethics Board and complied with the Declaration of Helsinki. All participants completed written informed consent prior to their involvement in the study.

### Study overview

The study consisted of a single experimental visit. Participants entered the laboratory after abstaining from caffeine, alcohol, and strenuous physical activity for a minimum of 12 h. Following voiding and anthropometric measurements, participants rested supine on a comfortable bed for the duration of the experiment. Baseline blood pressure measurements were taken from the left brachial artery (Model BPM-200, BpTRU, Coquitlam, BC). To ensure spontaneous breathing, respiratory excursions were monitored throughout the experiment using a piezoelectric respiration transducer (Model 1132 Pneumotrace II, UFI, Morro Bay, CA) placed around the mid-to-upper abdomen. As the depth of breathing can significantly influence MSNA (Seals et al., 1990), we also used the respiratory trace to measure within-participant peak-to-peak displacement (mV). Microneurography was used to record multi-unit MSNA from the right fibular nerve. Electrocardiography (Lead II) was used to record beat-to-beat heart rate. After instrumentation and familiarization with the vibration stimulus, a 10 min rest period was completed followed by six blocks of vibration stimulation. Three blocks of left hand palm and left foot sole vibration were completed (i.e., 6 total). The first vibration site was selected randomly for each participant, and followed by alternating blocks (e.g., hand → foot → hand). Each block of vibration consisted of 2 min of baseline followed by 2 min of vibration. To minimize any potential carryover effects, a 5 min rest separated each vibration block.

### Microneurographic recordings

Efferent postganglionic multi-unit MSNA was recorded from the right fibular nerve as previously described (Millar et al., 2013; Notay et al., 2016). Briefly, the path of the fibular nerve, posterior to the fibular head, was marked using palpation and transdermal electrical simulation (Grass Technologies, Warwick, RI). A 2 mΩ tungsten microelectrode (Frederick Haer, Brunswick, ME) was then inserted percutaneously into a fascicle of a motor nerve and manipulated until spontaneous, pulse synchronous, bursts of muscle sympathetic activity were detected audibly and visually. The raw MSNA signal was amplified (75,000–99,000x), bandpass filtered (700–2000 Hz), full wave rectified, and integrated (0.1 s time constant) (Nerve Traffic Analyzer, Model 662C-4; Absolute Design and Manufacturing Services, Salon, IA). Confirmation of the MSNA signal was made by verifying an absence of modulation during skin stroking or unexpected clapping, and an increase in response to an end-expiratory apnea.

### Cutaneous stimulation

**Figure 1:**
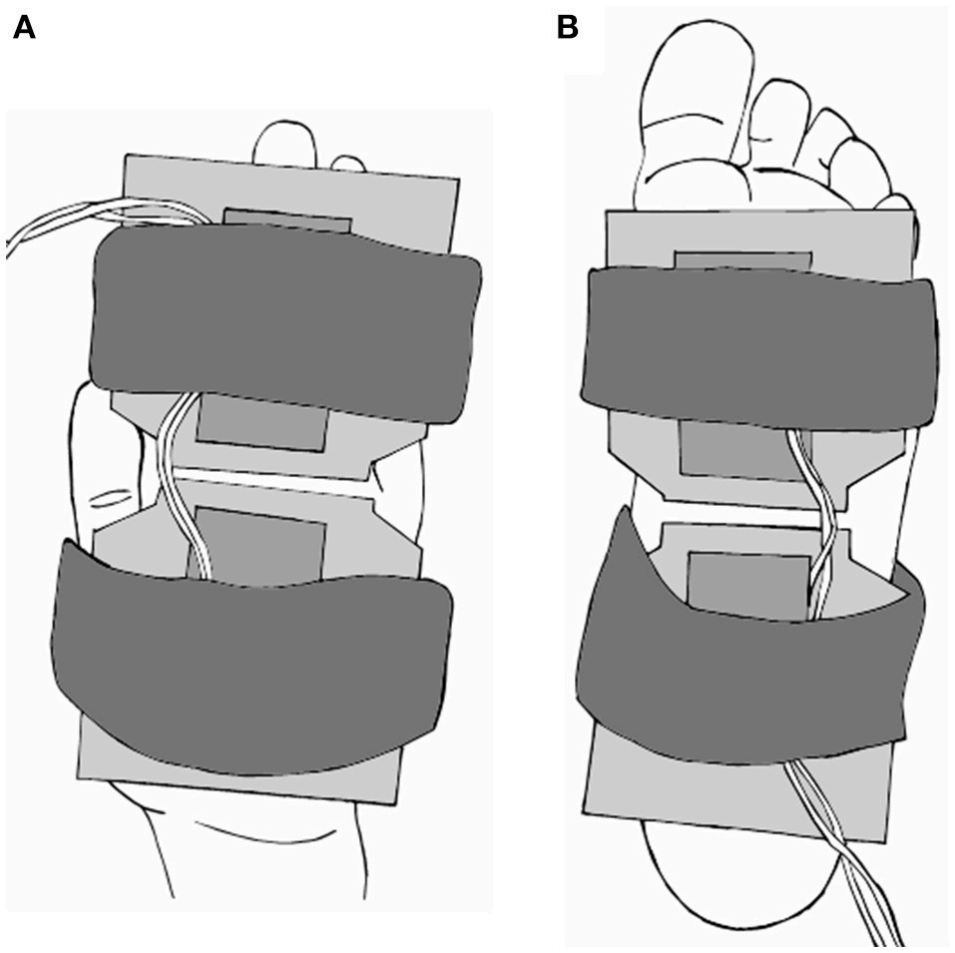
**Diagram of the vibration pads on the palm of hand (A)** and foot sole **(B)**. Two 7.5 cm by 7.5 cm pads were secured to each location with elastic straps, and positioned to maximize skin contact. Pads produced a mechanical vibration of 150 Hz, 220 μm peak-to-peak amplitude.

Low threshold cutaneous mechanoreceptors on the hand palm and foot sole were selectively activated using two custom-built vibration pads. Both vibration pads measured 7.5 cm by 7.5 cm and were controlled by a single amplifier to produce a 150 Hz, 220 μm peak-to-peak vibration. This stimulus was above perception threshold for all subjects, and known to generate a robust firing response across all four cutaneous afferent classes (Johansson et al., 1982). The interface of each pad was smooth dense foam. The pads were positioned to maximize glabrous skin contact on the hand and foot for each participant, while avoiding tendons in the wrist and ankle. On the palm of the hand, one pad covered the palm while the other primarily covered the fingers. On the foot sole, one pad was positioned to cover the heel and arch region while the second pad was positioned to cover the metatarsals (Figure 1).

### Data acquisition

All data were collected continuously at a sampling frequency of 1000 Hz, with the exception of the raw neurogram (10 kHz), and stored digitally using LabChart (PowerLab, ADInstruments, Colorado Springs, CO). The integrated neurogram was analyzed using custom LabView software (Millar et al., 2013, 2015; Notarius et al., 2014; Notay et al., 2016) and reported as MSNA burst frequency (bursts/min), burst incidence (bursts/100 heartbeats), and normalized mean burst amplitude (% of maximum) and area (% of maximum). As MSNA burst amplitude and area are influenced by the proximity of the recording electrode to the discharging fibers, we normalized the data as a percentage of the largest burst amplitude and area, respectively, within each 4 min trial. For each participant, all MSNA variables were calculated in whole numbers.

### Statistical analysis

Data are presented as mean ± *SD*. Heart rate and MSNA were calculated over the 2 min baseline and used to compare against the responses during the 2 min vibration period using paired *t*-tests. The three blocks of hand and foot vibration were averaged together as no significant differences in the change scores of each variable were detected using a one-way ANOVA. Based on prior evidence that stimulation of skeletal muscle afferents using passive stretch (muscle mechanoreflex) can produce significant but brief MSNA responses (Cui et al., 2006), we also sought to compare the baseline period against the first 10 s of vibration. Similar short epochs have been used to assess the rapid MSNA response to the onset of exercise (Greaney et al., 2014). Our group recently published data on the validity and reliability of measuring MSNA using short epochs and recommended the reporting of absolute burst count to reduce the risk of magnifying the error in such measurements (Notay et al., 2016). Paired *t*-tests were used similarly to analyze the MSNA burst count, burst frequency, burst incidence, and normalized mean burst amplitude and area between baseline and first 10 s of vibration. Significance was set at *p* < 0.05. All data were analyzed in GraphPad Prism (version 5.0c for Mac OS X, La Jolla, CA). Group MSNA data were reported to one decimal to provide greater insight into the magnitude changes.

## Results

**Figure 2:**
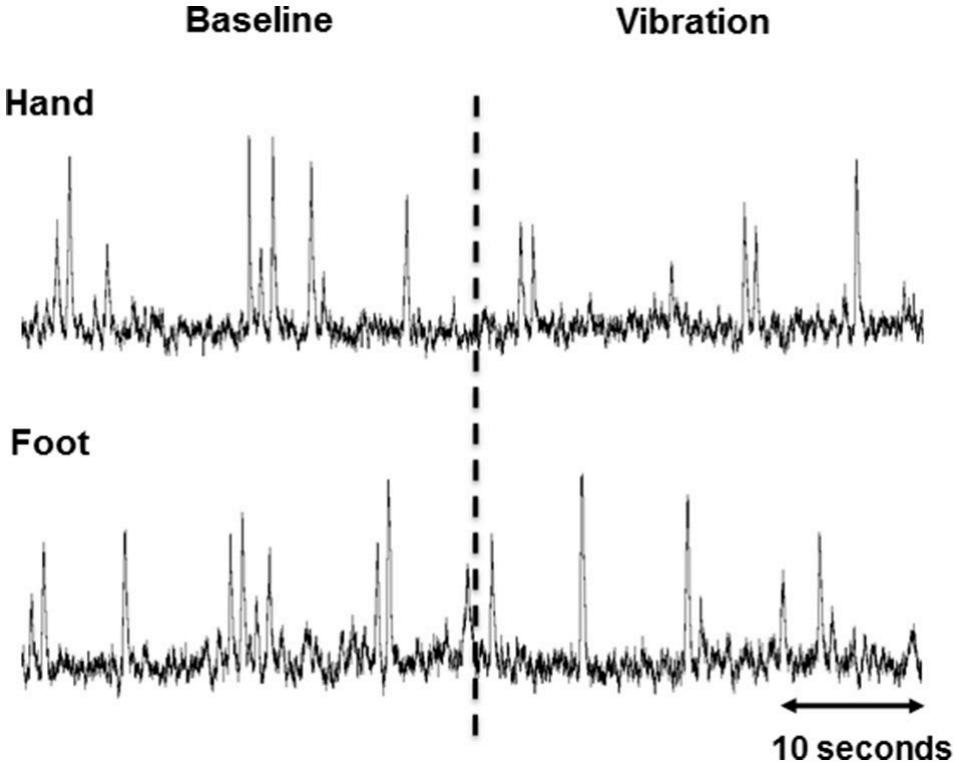
**Representative 60 s integrated muscle sympathetic nerve activity (MSNA) tracings from one participant during the end of baseline and beginning of vibration in the hand and foot**. The dashed line indicates the onset of vibration.

All 15 participants completed the study protocol. High-quality recordings of MSNA were obtained in 14 of 15 participants, with one being excluded from analysis due to a low signal-to-noise ratio. A representative tracing of the integrated neurogram from one participant during the end of baseline and onset of hand and foot vibration is presented in Figure 2.

**Figure 3:**
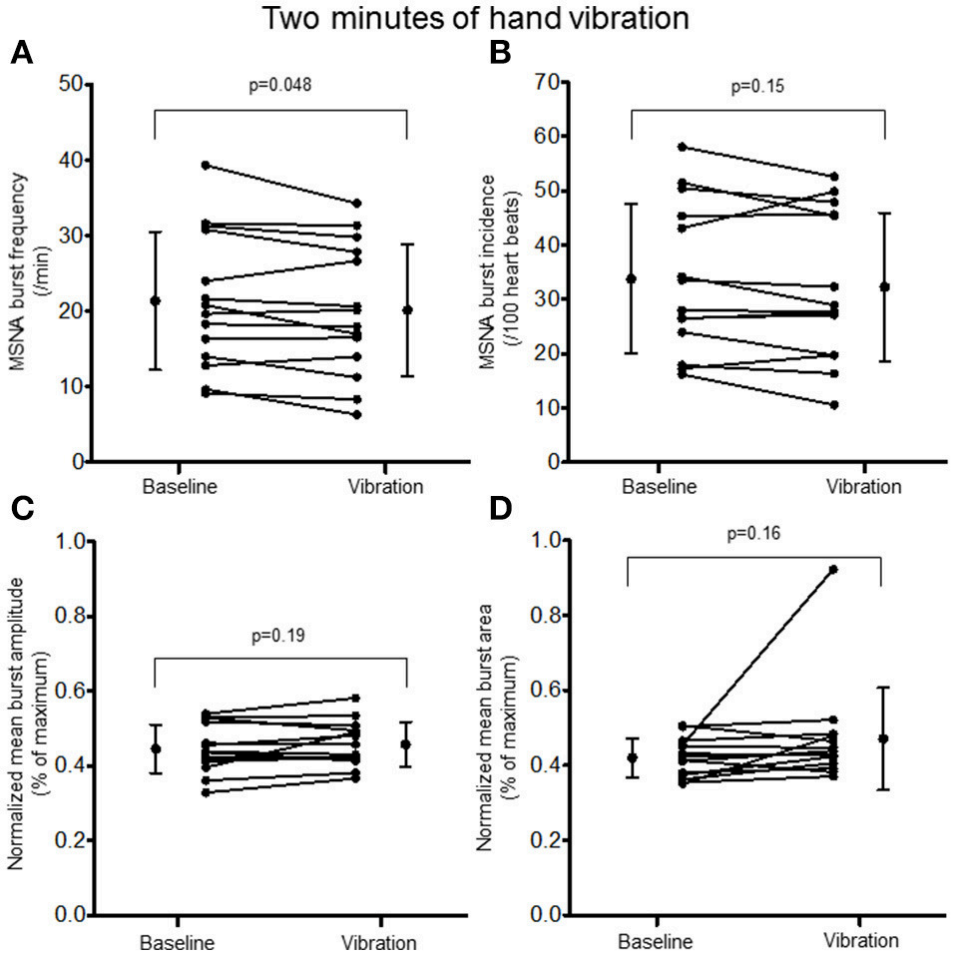
**Muscle sympathetic nerve activity (MSNA) burst frequency (A)**, burst incidence **(B)**, and normalized mean burst amplitude **(C)** and area **(D)** during 2 min of baseline rest and hand vibration.

**Figure 4:**
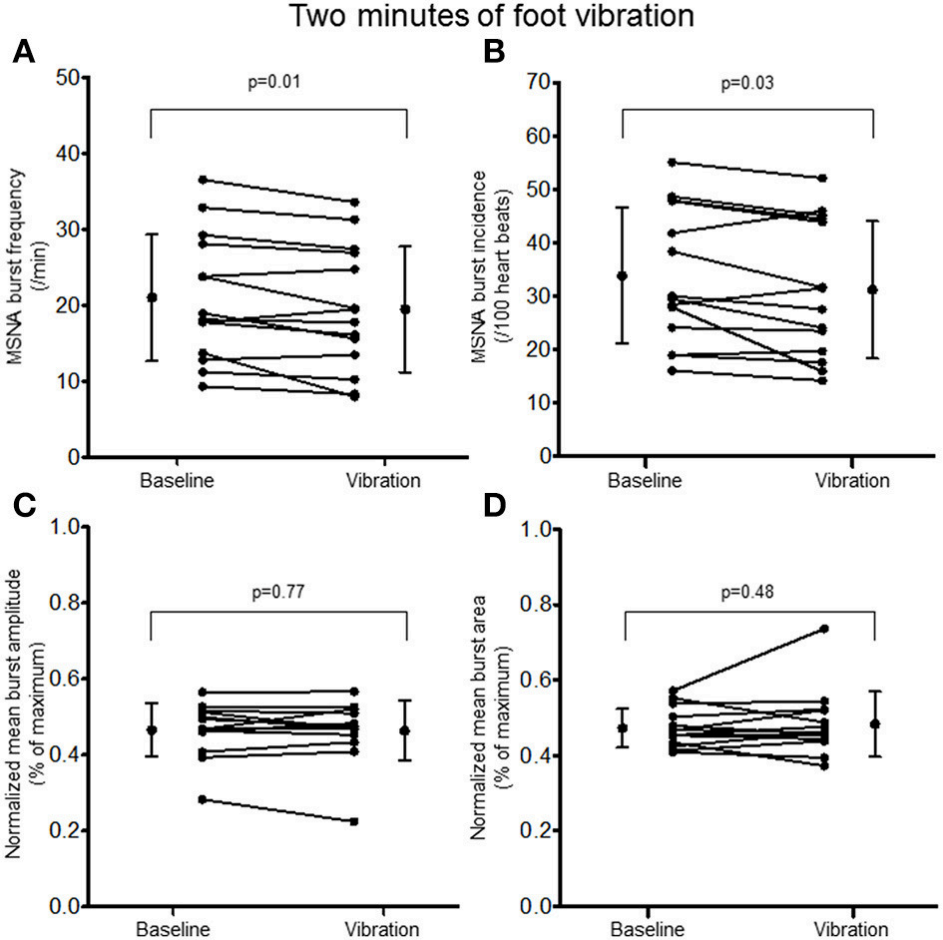
**Muscle sympathetic nerve activity (MSNA) burst frequency (A)**, burst incidence **(B)**, and normalized mean burst amplitude **(C)** and area **(D)** during 2 min baseline rest and foot sole vibration.

Two minutes of hand vibration decreased MSNA burst frequency (20.8 ± 8.9 vs. 19.3 ± 8.6 bursts/minute, Δ −8%, *p* = 0.048) in 10/14 participants, but did not alter burst incidence (32.8 ± 13.4 vs. 31.0 ± 13.4 bursts/100 heartbeats, *p* = 0.15), and normalized mean burst amplitude (0.45 ± 0.06 vs. 0.46 ± 0.06% of maximum, *p* = 0.19) or normalized mean burst area (0.42 ± 0.05 vs. 0.47 ± 0.14%, *p* = 0.16; Figure 3). Heart rate was slightly but consistently decreased during hand vibration (63 ± 8 vs. 62 ± 8 bpm, *p* = 0.001). Estimated breathing depth was unchanged (5.8 ± 2.7 vs. 5.7 ± 3.0 mV, *p* = 0.64). Similar to the hand, 2 min of foot sole vibration significantly decreased MSNA burst frequency (21.0 ± 8.3 vs. 19.5 ± 8.3 bursts/minute, Δ −8%, *p* = 0.01), as well as burst incidence (33.8 ± 12.7 vs. 31.2 ± 12.8 bursts/100 heartbeats, Δ −8%, *p* = 0.03; Figure 4). For foot sole vibration, reductions in MSNA burst frequency occurred in 11/14 participants. Normalized mean burst amplitude (0.47 ± 0.07 vs. 0.46 ± 0.08% of maximum, *p* = 0.77) and normalized mean burst area (0.47 ± 0.05 vs. 0.48 ± 0.09, *p* = 0.48) were unchanged (Figures 4C,D). Heart rate was unchanged from baseline throughout foot vibration (62 ± 8 vs. 62 ± 8 bpm, *p* = 0.75), as was estimated breathing depth (5.7 ± 3.0 vs. 5.3 ± 2.6 mV, *p* = 0.09).

**Figure 5:**
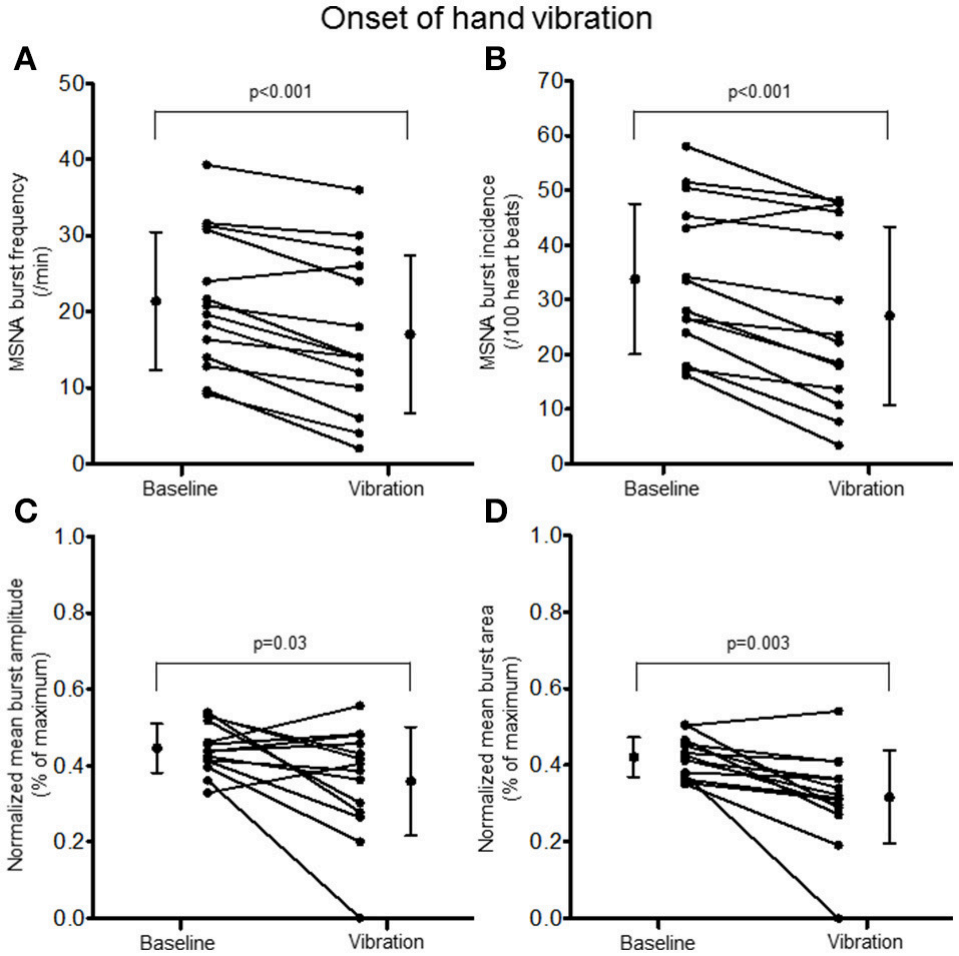
**Muscle sympathetic nerve activity (MSNA) burst frequency (A)**, burst incidence **(B)**, and normalized mean burst amplitude **(C)** and area **(D)** during 2 min of baseline rest and the onset (10 s) of hand vibration.

**Figure 6:**
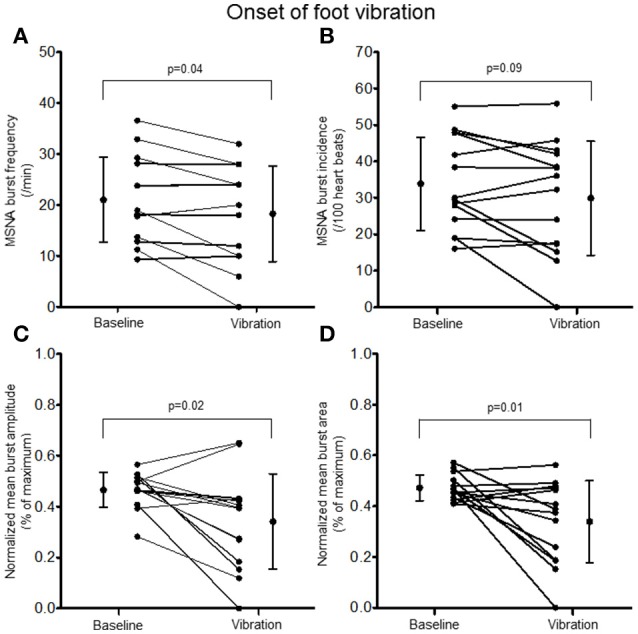
**Muscle sympathetic nerve activity (MSNA) burst frequency (A)**, burst incidence **(B)**, and normalized mean burst amplitude **(C)** and area **(D)** during 2 min of baseline rest and the onset (10 s) of foot sole vibration.

Examination of the MSNA response to the onset (first 10 s) of hand vibration demonstrated reductions in burst frequency (20.8 ± 8.9 vs. 17.0 ± 10.4 bursts/minute, Δ −25%, *p* < 0.001), burst incidence (32.8 ± 13.4 vs. 27.1 ± 16.3 bursts/100 heartbeats, Δ −24%, *p* < 0.001), normalized mean burst amplitude (0.45 ± 0.06 vs. 0.36 ± 0.14% of maximum, Δ −19%, *p* = 0.03), and normalized mean burst area (0.42 ± 0.05 vs. 0.32 ± 0.12%, Δ −25%, *p* = 0.003; Figure 5). Reductions in MSNA burst frequency occurred in 13/14 participants during the onset of hand vibration. Absolute burst count was reduced also from baseline (3.5 ± 1.5 vs. 2.8 ± 1.7 bursts/10 s, Δ −19%, *p* < 0.001) in 13/14 participants. In response to the onset of foot vibration, reductions in burst frequency (21.0 ± 8.3 vs. 18.3 ± 9.4 bursts/minute, Δ −16%, *p* = 0.04), normalized mean burst amplitude (0.47 ± 0.07 vs. 0.34 ± 0.19, Δ −27%, *p* = 0.15) and normalized mean burst area (0.47 ± 0.05 vs. 0.34 ± 0.16%, Δ −28%, *p* = 0.01) were noted, while a reduction in burst incidence approached significance (33.8 ± 12.7 vs. 29.9 ± 15.7, Δ −14%, *p* = 0.085; Figure 6). Reductions in MSNA burst frequency were found in 9/14 participants during onset of foot vibration. Absolute burst count was reduced also from baseline (3.5 ± 1.4 vs. 3.0 ± 1.6 bursts/10 s, Δ −13%, *p* < 0.05) in 9/14 participants. Heart rate was unchanged from baseline within the first 10 s of hand (63 ± 8 vs. 63 ± 9 bpm, *p* = 0.14) and foot (62 ± 8 vs. 62 ± 8 bpm, *p* = 0.14) vibration. Estimated breathing depth was also unchanged with hand (5.8 ± 2.7 vs. 5.6 ± 2.9 mV, *p* = 0.66) and foot (5.7 ± 3.0 vs. 5.0 ± 2.2 mV, *p* = 0.14) vibration.

## Discussion

The purpose of the present study was to examine the influence of low threshold cutaneous mechanoreceptor feedback from glabrous skin on the hand palm and foot sole on peripheral sympathetic outflow to skeletal muscle. The principal novel finding is that hand and foot vibration both produced small but consistent reductions in MSNA burst occurrence, with the largest reductions observed at the onset of vibration. These results provide support for a link between somatosensory afferent feedback from glabrous skin and autonomic efferent sympathetic outflow involved in cardiovascular regulation. Such tactile afferent feedback could play an important role in modulating efferent autonomic responses during postural challenges or exercise.

Neuroimaging studies demonstrate a convergence of somatosensory and baroreceptive afferents in forebrain structures (e.g., insula, ventral medial prefrontal cortex, anterior cingulate cortex) in both humans (Gray et al., 2009; Goswami et al., 2011) and monkeys (Zhang et al., 1999). These structures serve as part of the cortical autonomic network (CAN), a central circuit shown to integrate peripheral afferent feedback and to modulate efferent sympathetic and parasympathetic responses (Beissner et al., 2013; Shoemaker and Goswami, 2015). Goswami and colleagues demonstrated the involvement of the CAN in processing muscle afferent feedback through combined functional magnetic resonance imagining and electrical skin stimulation of forearm muscle afferents (Goswami et al., 2011). Subsequently, electrical stimulation of skeletal muscle afferents was shown to attenuate muscle sympathetic activation by ~4 bursts/minute during baroreceptor unloading using lower body negative pressure without altering heart rate, cardiac output, or mean arterial pressure (Goswami et al., 2012). No changes in MSNA were evident during combined skeletal muscle somatosensory stimulation and an expiratory apnea (Goswami et al., 2012), suggesting that muscle afferents do not converge with chemoreceptor inputs. Whether cutaneous afferents are associated similarly with the CAN is unclear as this previous work anesthetized the skin to limit their influence (Goswami et al., 2011, 2012). In monkeys, insular neurons are responsive to changes in both blood pressure and nociceptive pinching, a finding that supports the primate insular cortex as a integration site for cardiovascular regulation (Zhang et al., 1999). In humans, non-painful electrocutaneous stimulation delivered to the hand dorsum across the cardiac cycle modulated activity within the insula, in addition to the amygdala and pons (Gray et al., 2009). Cutaneous stimuli delivered during early systole inhibited increases in blood pressure reflecting the central integration of cutaneous and baroreceptor input and a phasic efferent response. However, while it is evident that somatosensory inputs can influence autonomic activity, the direct role of cutaneous mechanoreceptors remains confounded by skin anesthetization (Goswami et al., 2011, 2012) or the use of noxious (Nordin and Fagius, 1995) or startle stimuli (Donadio et al., 2007).

Cutaneous afferents are responsible primarily for providing the central nervous system with tactile and proprioceptive feedback; information critical for the exploration and manipulation of objects in the hands and maintenance of posture and balance over the feet (Kavounoudias et al., 1998; Yau et al., 2016). Additionally, the current data show that vibration of low threshold cutaneous afferents in the hand palm and foot sole can also modulate efferent sympathetic drive at rest. In line with our primary hypothesis, 2 min of hand and foot vibration both reduced MSNA burst frequency by ~8%. Our results are comparable to a 9% reduction in MSNA burst frequency reported following 5 min of transcutaneous electrical stimulation (80 Hz, 200 μs pulse width, 3 s on–3 s off) without muscular contraction (i.e., sensory stimulation) in heart failure patients (Labrunée et al., 2013). Further, the reductions in MSNA burst frequency in the present study were largely consistent between participants arguing against a role for arousal in mediating the sympathetic responses; electrical finger shocks evoke either a reduction or increase in MSNA in approximately equal proportions of participants (i.e., positive and negative responders), a response that is consistent within an individual over time (Donadio et al., 2002a,b). The functional importance of a sympathoinhibitory low threshold mechanoreceptor cutaneous reflex is unclear. Modest reductions in MSNA with somatosensory stimulation (~4–6 bursts/min) can occur independent of changes in blood pressure, heart rate, or respiration (Goswami et al., 2012; Labrunée et al., 2013). It is also important to consider that isolated stimulation of peripheral afferent reflexes at rest may be buffered by engagement of the arterial baroreflex (Cui et al., 2006), and that the reflex gain and physiological significance may be larger during a stress or exercise challenge. In support, skeletal muscle somatosensory stimulation did not alter MSNA at rest unless combined with baroreflex unloading (Goswami et al., 2012). Nevertheless, our findings suggest that feedback from cutaneous mechanoreceptors may contribute to the integrated control of central sympathetic outflow.

The vibration stimulus (150 Hz, 220 μm) used in the present experiment was chosen to selectively activate low threshold mechanoreceptor afferents without the potentially confounding effects of evoking a noxious or arousal response. The stimulus was above perceptual threshold for all participants, in agreement with established vibrotactile thresholds (Bolanowski et al., 1988; Strzalkowski et al., 2015b; Mildren et al., 2016). High frequency stimuli (above ~30 Hz) has been shown to activate predominantly fast adapting cutaneous afferents, however, at the high amplitude used in the present study, afferent firing is expected across all four afferent classes (Johansson et al., 1982). Fast adapting type I (FAI) afferents are the most abundant class of cutaneous afferent in the hands (Johansson and Vallbo, 1980) and feet (Strzalkowski et al., 2015a) and FAI afferent feedback may therefore have the largest impact on the observed modulation of MSNA. Lower limb FAI afferents have been shown previously to have the largest impact on modulating motor unit excitability in the lower (Fallon et al., 2005) and upper limbs (Bent and Lowrey, 2013) compared to other afferent classes. The aim of the present study was not to investigate specific cutaneous afferent classes *per-se*, however, due to the abundance of FAI afferents in the glabrous skin and the high frequency vibration applied, FAI afferent activity may have a proportionately higher impact on the observed changes. The abundance and sensitivity of FAI afferents to light touch and vibration (Johansson et al., 1982; Strzalkowski et al., 2015a) make them ideally suited to provide feedback for efferent autonomic regulation, although future studies are needed to evaluate the influence of different tactile stimuli and combinations of cutaneous afferent feedback on MSNA.

There are several considerations to acknowledge in interpreting the present findings. First, we studied a young healthy adult population and our results may not generalize to older populations. Tactile sensitivity and cutaneous afferent firing capabilities decrease with age (Deshpande et al., 2008), which may limit the effects on efferent sympathetic responses. Second, the physiological significance of a small reduction in MSNA is unclear with future work needed to establish whether these neural changes impact hemodynamic measures. Third, we cannot rule out definitively that our observations were not the result of an arousal-mediated autonomic, motor, or respiratory response evoked by the vibration. However, participants underwent pre-study familiarization, were given an auditory countdown to the initiation of the non-painful vibration stimulus, and demonstrated small or no change in heart rate and estimated breathing depth. Fourth, it is possible that the vibration stimulus activated muscle spindle afferents sensitive to tendon vibration (Burke et al., 1976). To limit tendon contact and spindle firing, the vibration pads were located on the palm of the hand and foot sole and the vibration amplitude (220 μm) was lower than reported spindle afferent firing thresholds (Ia ~225 μm, II ~260 μm; Fallon and Macefield, 2007). Further, prior work has demonstrated that electrical stimulation of group I and II skeletal muscle afferents while undergoing cutaneous afferent blockade had no effect on resting MSNA (Goswami et al., 2012). Finally, the area of skin targeted in the present study was small (<56 cm^2^), and consisted of a single vibration stimulus (150 Hz at 220 μm). More robust and natural forms of tactile feedback applied in different postural contexts may evoke larger changes in MSNA. Despite these considerations, the present data support a role of cutaneous afferent feedback in modulating efferent sympathetic outflow to skeletal muscle.

In conclusion, the present findings suggest a contributory role for cutaneous somatosensory afferent feedback in the regulation of efferent sympathetic outflow to skeletal muscle. Vibrotactile stimulation of low threshold cutaneous mechanoreceptors from the glabrous skin of the hand palm and foot sole decreased efferent sympathetic outflow to skeletal muscle in young healthy adults, with the largest responses in MSNA burst frequency and amplitude observed at the onset of vibration. Future work is required to determine the mechanisms responsible for these responses and the functional hemodynamic implications at rest and during stress.

## Author contributions

NS, LB, and PM conceived and designed research; NS, AI, and PM performed experiments; NS analyzed data; NS, and PM interpreted results; NS, AI, and PM prepared figures and drafted manuscript; NS, AI, LB, and PM edited and revised manuscript; NS, AI, LB, and PM approved final version of manuscript.

## Funding

This research was supported by a Natural Science and Engineering Research Council (NSERC) of Canada Discovery Grant (PM; #1256447; LB; #400723) and the Canada Foundation for Innovation (PM; #34379). AI was supported by a Canadian Institutes of Health Research (CIHR) Fredrick Banting and Charles Best Canada Graduate Scholarship.

### Conflict of interest statement

The authors declare that the research was conducted in the absence of any commercial or financial relationships that could be construed as a potential conflict of interest.
